# Coexistence of Ankylosing Spondylitis and Löfgren's Syndrome

**DOI:** 10.1155/2014/747698

**Published:** 2014-03-11

**Authors:** Senol Kobak, Fidan Sever, Oya Sivrikoz, Ahmet Karaarslan

**Affiliations:** ^1^Department of Rheumatology, Faculty of Medicine, Sifa University, 35100 Bornova, Izmir, Turkey; ^2^Department of Chest Diseases, Faculty of Medicine, Sifa University, 35100 Bornova, Izmir, Turkey; ^3^Department of Pathology, Faculty of Medicine, Sifa University, 35100 Bornova, Izmir, Turkey; ^4^Department of Orthopedics, Faculty of Medicine, Sifa University, 35100 Bornova, Izmir, Turkey

## Abstract

A 46-year-old male patient diagnosed with ankylosing spondylitis presented to our polyclinic with complaints of pain, swelling, and limitation in joint mobility in both ankles and erythema nodosum skin lesions in both pretibial sites. The sacroiliac joint graphy and the MRI taken revealed active and chronic sacroiliitis. On the thorax CT, multiple mediastinal and hilar lymphadenopathies were reported. Mediastinoscopic excisional lymph node biopsy was taken and noncalcified granulomatous structures, lymphocytes, and histiocytes were determined on histopathological examination. The patients were diagnosed with ankylosing spondylitis, sarcoidosis, and Löfgren's syndrome. NSAIDs, sulfasalazine, and low dose corticosteroid were started. Significant regression was seen in the patient's subjective and laboratory assessments.

## 1. Introduction

Sarcoidosis is a systemic disease characterized by the involvement of multiple tissues and organs and a noncalcified granulomatous reaction, which is not well understood [1]. Löfgren syndrome is an acute sarcoidosis presentation characterized by arthritis/arthralgia, erythema nodosum (EN), and bilateral hilar lymphadenopathy. Although its pathogenesis in not clear, there appears to be a cellular immune system activation and a nonspecific inflammatory response against some genetic and environmental factors [2]. Th1-lymphocyte and macrophages caused by proinflammatory cytokines induce the inflammatory cascade and the formations of granulomas occur as a result of tissue permeability, cellular influx, and local cell proliferation [3]. The indispensable pathological finding of sarcoidosis is noncalcified epitheloid cellular granulomas [4]. Different prevalence, clinical findings, and course of disease in different races and ethnic groups suggest that sarcoidosis is a heterogeneous disease [5]. The disease is more prevalent in women and develops after 40 years of age. Sarcoidosis is a chronic granulomatous disease that may present with different clinical findings. The disease most frequently presents with bilateral hilar lymphadenopathy, infiltrations in the lungs, and skin and eye lesions. It may mimic a number of primary rheumatic diseases (connective tissue diseases, vasculitis, and spondyloarthritis) and/or develop concomitantly to these [6]. Locomotor involvement is determined to be 15–25%. The sacroiliac joint involvement typical in ankylosing spondylitis (AS) is rarely observed in sarcoidosis. In this paper, we report a case of ankylosing spondylitis coexisting with Löfgren's syndrome.

## 2. Case Report

**Figure 1:**
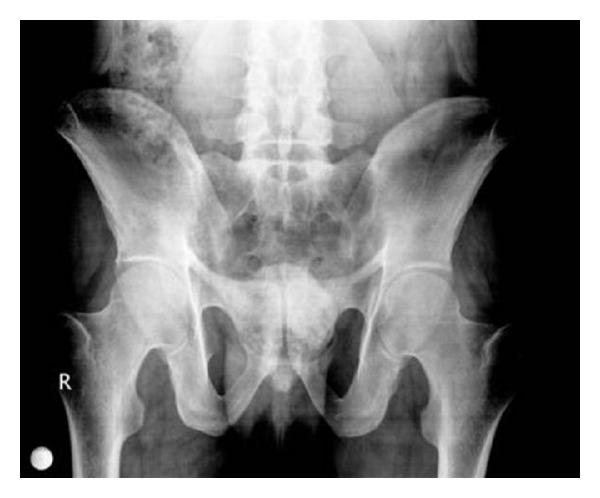
Sacroiliac joint graphy showed bilateral chronic sacroiliitis.

**Figure 2:**
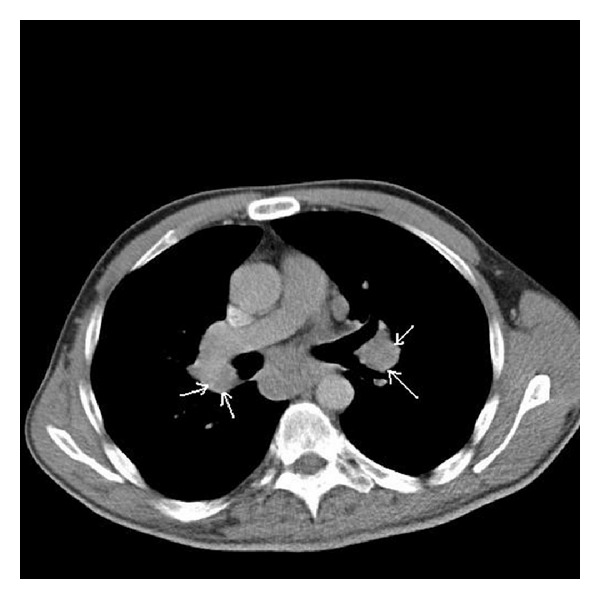
Thorax CT showed multiple mediastinal and hilar lymphadenopathies.

A 46-year-old male patient presented to our polyclinic with complaints of pain, swelling, and limitation in joint mobility in both ankles and erythema nodosum skin lesions in both pretibial sites. The history of the patient revealed that he has an AS diagnosis for 15 years and that he did not take his medicine or go to controls for the past 5 years. Upon questioning, he described inflammatory lower back pain, morning stiffness lasting more than one hour, pain in the heels, and fatigue. On physical examination, it was determined that the posture reflected advanced AS and bilateral ankle arthritis; significant limitation in the motion of the neck was observed, and bilateral FABERE and FADIR were positive. At the measurements performed, Schöber test was measured as 2 cm, chest expansion as 1.5 cm, hand to floor distance as 26 cm, occiput to wall distance as 6 cm, and jaw to sternum distance as 2 cm. BASDAI was determined to be 7.8 cm and BASFI to be 4.5 cm. At laboratory examination, WBC was determined as 10800/uL, Hgb as 11.6 g/dL, Htc as 37.4%, Plt as 465000/uL, fasting blood sugar as 90 mg/dL, urea as 14 mg/dL, creatinine as 0.82 mg/dL, AST as 66 U/L, ALT as 70 U/L, T. protein as 7.1 g/dL, albumin as 4.3 g/dL, serum ACE as 83 U/L (normal: 8–52 U/L), and serum amyloid A as 923 mg/L (normal: <10 mg/L). C-reactive protein was 12.81 mg/dL (normal: 0–0.5 mg/dL) and ESR was 102 mm/h (normal: <30 mm/h). Routine urinalysis was normal. Hepatitis serology was tested and anti-HCV, HBsAg, and anti-HIV were negative. Thyroid function tests were normal. During serological tests, RF, ANA, and anti-CCP were detected to be negative. HLAB-27 was positive. The sacroiliac joint graphy and the MR taken revealed chronic sacroiliitis as well as findings of bone oedema at the bilateral sacral and iliac wings (Figure 1). Cervical, thoracic, and lumbar graphs were consistent with advanced stage AS. The abdominal USG was normal, except for grade 1 hepatosteatosis. The lung graphy revealed hilar fullness. On the thorax CT, multiple mediastinal and hilar lymphadenopathies with a maximum diameter of 35 mm and, at the left lung subpleural site, nodular density areas with a maximum diameter of 1.4 cm were reported (Figure 2). As a result, a thoracic diseases specialist was consulted and a PET was made. On the thorax CT, no activity involvement was observed in the LAP and the nodules; they were reported to be of benign nature. A bronchoscopy was performed; no endobronchial lesion was detected, and no malign cell was determined in the BAL fluid taken. After consulting with a thoracic surgeon, a mediastinoscopic excisional lymph node biopsy was taken. On histopathological examination, noncalcified granulomatous structures, lymphocytes, and histiocytes were determined. The microbiological examinations for tuberculosis (including tissue analysis by PCR) came back negative. In addition, a rectum biopsy was taken for secondary amyloidosis; the Congo-red staining was reported to be negative. Based on clinical laboratory and radiological and histological examinations, the patients were diagnosed with ankylosing spondylitis, sarcoidosis, and Löfgren's syndrome. NSAIDs, sulfasalazine, and low dose corticosteroid were started. At month 1 of the treatment, a significant regression was seen in the patient's subjective and laboratory assessments. The patient, who was generally in a good condition, continues to be monitored at the polyclinic.

## 3. Discussion

Sarcoidosis is a chronic, granulomatous, and multisystemic disease. Löfgren's syndrome is an acute sarcoidosis manifesting as a combination of arthralgia/arthritis, bilateral hilar lymphadenopathy, and erythema nodosum. It usually has a good prognosis, with spontaneous remission [7]. In this paper, we are reporting a male case with a diagnosis of AS, who had sarcoidosis coexisting with Löfgren's syndrome, as determined by laboratory, radiological, and histopathological assessments.

AS coexisting with sarcoidosis has been reported in the literature in approximately 18 patients and may present in two different ways: sarcoidosis developing in patients with AS and/or sacroiliitis developing as a result of sarcoidosis. In patients with AS, development of sarcoidosis has been reported in the literature [8]. All these patients were HLA-B27-positive and first sacroiliitis developed, which was later followed by lung involvement related sarcoidosis. The diagnoses of all these cases were made by lung biopsies revealing noncalcified granulomas. Similarly, after administration of anti-TNF-alpha in patients with AS, emergence of sarcoidosis was reported [9]. It is believed that sarcoidosis is triggered by different mediating infectious agents, following suppression of TNF-alpha cytokine, which plays an important role in the noncalcified granuloma formation seen in sarcoidosis. However, in another paper, it was reported that infliximab may be an effective and safe therapy in a patient diagnosed with both AS and sarcoidosis [10]. This result was attempted to be explained with the common etiological and pathogenetic mechanisms of both diseases. Evidence for this coexistence was provided with the fact that both diseases cause uveitis and sacroiliitis, with the predominance of CD4+ T-lymphocytes and the presence of common agents (like* Propionibacterium acnes*) that are controversial in the pathogenesis. Sarcoidosis may mimic AS by causing sacroiliitis [11]. In the literature, 4 cases have been reported where sarcoidosis involved the sacroiliac joint. A sacroiliac joint biopsy was done and diagnosis was made by showing noncalcified granulomas. In the genetic analysis of these 4 patients, HLA-B27 was determined to be negative, while HLA types associated with sarcoidosis (B8, B13, B35, and A9) were detected. In a recently performed cross-sectional study, radiological sacroiliitis was detected in 6.5% of patients with sarcoidosis, compared to 1.9% of the normal population. These data do not support a real coexistence between SpA and sarcoidosis; however, 1 in 4 patients with sacroiliitis was found to be HLA-B27-positive. In another study, a relationship was established between isolated pulmonary sarcoidosis and HLA-B27, which might suggest a real coexistence [12]. However, when considering the high prevalence of both diseases (1/1000 for AS and 0.4–64/1000 for sarcoidosis), coexistence seems rather sporadic. AS and sarcoidosis are two different diseases and it is clear that they do not have a common basis. MHC class I coding alleles are causing a genetic predisposition to AS, whereas sarcoidosis is associated with MHC class II coding alleles [13]. While HLA-DR5 is significantly higher in German patients with sarcoidosis, HLA-B8 has been associated with acute sarcoid arthritis and spontaneous remission. Our case has also been determined to be HLA-B27-positive, but genetic tests associated with sarcoidosis could not be carried out. The presence of typical HLA alleles for both diseases suggests that these two independent diseases primarily have a coincidental coexistence.

As a result, we are reporting the coexistence of AS with sarcoidosis. Prevalence data appear to be important arguments for coincidence, rather than absence, of a genetic relationship or a response to treatment. New, multicenter studies are required to gain more insight into these issues.
